# Estimating In Vitro Protein Digestion and Protein Digestibility Corrected Amino Acid Score of Chicken Breasts Affected by White Striping and Wooden Breast Abnormalities

**DOI:** 10.3390/foods13010159

**Published:** 2024-01-02

**Authors:** Yanee Srimarut, Apinya Phanphuet, Thanatorn Trithavisup, Wachiraya Rattanawongsa, Rattaporn Saenmuangchin, Annop Klamchuen, Yuwares Malila

**Affiliations:** 1 Food Biotechnology Research Team, National Center for Genetic Engineering and Biotechnology, Pathum Thani 12120, Thailand; yanee.sri@biotec.or.th (Y.S.); apinya.pha@ncr.nstda.or.th (A.P.); kamonrat26@gmail.com (T.T.); 2 Nanocharacterization Research Team, National Nanotechnology Center, Pathum Thani 12120, Thailand; wachiraya.rat@ncr.nstda.or.th (W.R.); rattaporn.sae@nanotec.or.th (R.S.); annop@nanotec.or.th (A.K.)

**Keywords:** chicken meat, growth-related myopathy, in vitro protein digestion, protein digestibility corrected amino acid score

## Abstract

An understanding regarding impacts of growth-related myopathies, i.e., white striping (WS) and wooden breast (WB), on the quality of dietary protein from cooked chicken breast is still limited. This study aimed at comparing protein content and in vitro protein digestion and estimating the in vitro protein digestibility corrected amino acid score (PDCAAS) of cooked chicken meat exhibiting different abnormality levels (i.e., normal, WS, and WS + WB). The results show that the WS + WB samples exhibited lower protein content, greater cooking loss, and greater lipid oxidation than those of normal samples (*p* < 0.05). No differences in protein carbonyls or the myofibril fragmentation index were found (*p* ≥ 0.05). Cooked samples were hydrolyzed in vitro using digestive enzyme mixtures that subsequently mimicked the enzymatic reactions in oral, gastric, and intestinal routes. The WS + WB samples exhibited greater values of free NH_2_ and degree of hydrolysis than the others at all digestion phases (*p* < 0.05), suggesting a greater proteolytic susceptibility. The in vitro PDCAAS of the WS + WB samples was greater than that of the other samples for pre-school children, school children, and adults (*p* < 0.05). Overall, the findings suggest that the cooked chicken breast with the WS + WB condition might provide greater protein digestibility and availability than WS and normal chicken breasts.

## 1. Introduction

The issue of future food scarcity has raised concern worldwide. This is because the global population is projected to reach 9.7 billion by 2050. Despite limited land, water, and other natural resources, more nutritious foods, particularly food proteins, are required to feed the massive population [1]. Although alternative proteins, e.g., plant-based, insect-based, microbial-derived, and animal cell-based proteins, have been under extensive focuses [2,3,4,5], it is anticipated that chickens will continue to be an important source of food protein in the coming decades. This is based upon the fact that chickens offer high-quality protein at an affordable price across all socioeconomic classes [6].

Poultry meat has been in high demand for several decades. To meet consumer demand, a breeding selection program focusing on high production performance has been established. As a result, commercial broilers can reach their market weight within 6 weeks of age [7]. However, an increased prevalence of meat abnormalities known as white striping (WS) and wooden breast (WB) have been detected among commercial broilers in the past decade [8,9]. The WS abnormality is classified based on the occurrence of white lines on the surface of the meat. The more lines there are and the thicker they are, the more severe the WS myopathy is [9,10,11]. As for WB meat, the meat exhibits palpable hardness and pale ridges mainly on the caudal region of the breast [9,10,12]. Increased WB severity is characterized by extended ridges covering the whole breast, often accompanied by viscous fluid and hemorrhages [9,10]. The abnormalities may occur independently or in combination (WS + WB), with an average prevalence rate of approximately 70% per flock [9,10,13]. Previous studies pointed out a close association between the occurrence and severity of the myopathies and the accelerated growth rate of commercial broilers [8,9,10,14], leading to the recognition of these issues as “growth-related myopathies.” Differential visual appearance of growth-related myopathies significantly reduced consumer acceptance of the meat [15]. In addition, the breast meat consistently demonstrated low water-holding capacity and a tough texture [8,11,16]. Hence, the severely affected meats are usually downgraded, leading to an economic loss in poultry industry [8,17]. In this regard, the presence of WS and WB has raised significant concern within the industry.

In terms of protein quality, several studies consistently addressed the changes in muscle protein composition due to growth-related myopathies. The changes included increased collagen content, particularly insoluble collagen [16,18], and deviated profiles of myofibrillar and sarcoplasmic proteins [19,20,21,22,23]. Dalle Zotte et al. [24] also reported altered amino acid profiles within raw abnormal meat. In addition, WS and WB meat showed decreased total protein content [11,16,18,21] and reduced protein extractability [19,25]. Nonetheless, it remains unclear whether such changes are the causes or outcomes of growth-related myopathies. As per nutritive values and health effects, direct investigation of those aspects is relatively limited. The abnormal meat is still considered edible for human consumption and does not lead to any acute harmful impacts on human health. However, a recent report by Soglia et al. [26] indicated an increase in free amino acids in raw WB meat. Their findings suggest that muscular protein breakdown occurs as a result of myodegeneration within the affected muscle. Those free amino acids could be lost in dripping fluids during thawing and cooking processes, altering the nutritional quality of the meat.

Additionally, increases in protein carbonyl and thiobarbituric acid reactive substance (TBARS) values, indicating protein and lipid oxidation, respectively, were addressed in affected chicken breasts [23,27,28]. Oxidation may modify the target sites on protein molecules for proteases, hence lowering protein digestibility [29]. Moreover, accumulated evidence began to point out the potential health risk of consuming diets with excessive oxidative products [30]. Those oxidized molecules may induce redox imbalance in the epithelial cells of the consumer’s digestive tract [31]. Long-term exposure to excessively oxidized diets may induce cellular inflammation and exacerbate other deleterious physiological conditions [32]. Recently, we conducted in vitro protein digestion for chicken breasts exhibiting severe WB condition and found greater protein digestion in the WB samples compared with the non-WB ones [33]. This was potentially due to muscle degeneration that was consistently observed in the WB meat [26]. On the contrary, information about WS myopathy and the combination of WS and WB is still limited. Breasts with mild to moderate WS myopathy appear to be “a new norm” for modern poultry meat [34]. Hence, there is a high possibility that WS breasts can be consumed.

In general, the quantity of protein in a food is primarily reported in terms of the true nitrogen content. However, the amount of crude protein alone does not directly reflect dietary protein quality in the food item [35]. Dietary protein quality can be defined based on the extent to which amino acid composition matches individuals’ metabolic requirements [35]. Those include digestibility, bioavailability, and metabolic utilization of the protein in the body [35]. In 1989, the FAO/WHO recommended the protein digestibility corrected amino acid score (PDCAAS) as the most suitable method for evaluating dietary protein quality [35]. The PDCAAS value accounts for essential amino acid constituents of dietary protein and true fecal nitrogen digestibility. Determining the PDCAAS in vivo requires high costs, long experimental duration, and ethical objections to animal use [36]. In this regard, an application of in vitro PDCAAS as an alternative method was previously examined, and a strong correlation between the in vitro and in vivo PDCAAS was reported [37]. Therefore, the objective of the present study was to determine the effects of growth-related myopathies, i.e., WS and WS + WB, on the in vitro protein digestion of cooked chicken meat and to estimate the in vitro PDCAAS values.

## 2. Materials and Methods

### 2.1. Samples

Chicken breast (*pectoralis major*) samples from commercial broilers were purchased and collected from a local slaughterhouse (Pathum Thani, Thailand). All samples were from one slaughtering batch to minimize other confounding effects. The meat was classified as “normal,” “WS,” or “WS + WB” based on classification criteria specified in the study of Malila et al. [11]. In brief, the WS samples were classified based on the appearance of white lines running parallel to the muscle fibers on the superficial area of the breast. The numbers of lines needed to be more than 40 or up to 5 white lines showing a thickness of 1.0 mm to 1.9 mm. The condition of WB was characterized based on palpation. The entire breast needed to be significantly rigid. The classification was carried out by one trained staff member to minimize other confounding factors. It is worth noting that the samples with the WS condition alone and with WS + WB would fall within the groups of “moderate WS” and “moderate WS + WB,” as classified in the study by Malila et al. [11].

A total of 30 chicken breasts (*n* = 10 for each treatment, 4 raw, 6 cooked) were used in this study. In each treatment group, four pieces of the samples were proceeded for analyses of protein [38], oxidation of protein and lipids [27], and the myofibril fragmentation index (MFI) [33]. The other six samples in each treatment group were subjected to cooking. The cooked samples were then subjected to protein determination [38], amino acid composition profiling, and in vitro protein digestion.

### 2.2. Cooking Procedure

Chicken breast samples were cooked using a water immersion method [11]. In brief, each breast sample was individually vacuum-packed in a polyethylene bag and incubated at 95 °C until its core temperature reached 80 °C. The samples were subsequently cooled to 15 °C by immersing in an ice water bath. Each sample was left to rest at 4 °C for 2 h before grinding using a household blender. Cooking loss was calculated as the difference in weight, expressed as a percentage, before and after the meat was cooked.

### 2.3. Oxidation of Protein

Oxidation of protein was determined based on protein carbonyl content [33]. In brief, 1 g of chopped raw sample was homogenized with 10 mL of 0.15 M KCl for 30 s. One hundred microliters of the homogenate were mixed with 1 mL of ice-cold acetone to precipitate the protein. The resulting protein was collected via centrifugation at 3500× *g* for 2 min. The pellet was then resuspended in 0.4 mL of 5% SDS with an incubation of 100 °C for 10 min. Two hundred microliters of protein solution were then used for the determination of protein carbonyls using a protein carbonyl assay kit (Sigma-Aldrich, St. Louis, MO, USA) following the manufacturer’s protocol. Protein carbonyl content was calculated using a millimolar extinction coefficient at 375 nm of 22 mM^−1^ cm^−1^ and was expressed as nmol per mg protein.

### 2.4. Oxidation of Lipids

Oxidation of lipids was analyzed as thiobarbituric acid reactive substances (TBARS) [27]. In brief, 2 g of meat samples were homogenized with 10 mL of TBARS reagent (26 mM thiobarbituric acid, 0.92 M trichloroacetic acid (TCA) and 0.25 M HCl) using an ULTRA-TURRAX T25 homogenizer (IKA Werke, Staufen, Germany) at 11,000 rpm for 30 s. The mixture was then incubated in a boiling bath for 10 min, followed by cooling with running tap water and centrifugation at 3600× *g* for 25 min. Absorbance at 532 nm of the resulting supernatant was measured against a reagent blank. A standard curve of 1, 1, 3, 3, tetraethoxypropane was constructed and used for TBARS value calculation. The result was expressed as μmol malondialdehyde per kg sample.

### 2.5. Myofibril Fragmentation Index

The MFI of raw samples was analyzed as per Hopkins et al. [39]. Briefly, half a gram of the sample was homogenized at refrigeration temperature in 30 mL of cold MFI buffer (25 mM potassium phosphate buffer (pH 7.0), 0.1 M KCl, 1 mM EDTA, and 1 mM sodium azide) using an ULTRA-TURRAX T25 homogenizer (IKA Werke) equipped with an S25KV-18G dispersing probe (IKA Werke) at a speed of 13,500 rpm. The homogenization was performed for a total of 2 min with two cycles of 30 s on and 30 s rest. The homogenate was then filtered through two-layer gauze to remove any connective tissues. Subsequently, the filtrate was centrifuged at 1000× *g* for 10 min. The resulting pellet was collected and resuspended in 20 mL of cold MFI buffer. The extraction was conducted twice. The final pellet was resuspended in 10 mL MFI buffer and its protein concentration was determined using a bicinchoninic acid assay. The suspension was diluted with cold MFI buffer to a protein concentration of 0.5 mg/mL and a final volume of 2 mL. The absorbance of the suspension was measured at 540 nm using MFI buffer as a blank. The MFI was calculated by multiplying the average absorbance by 150 [40].

### 2.6. In Vitro Protein Digestion

**Figure 1:**
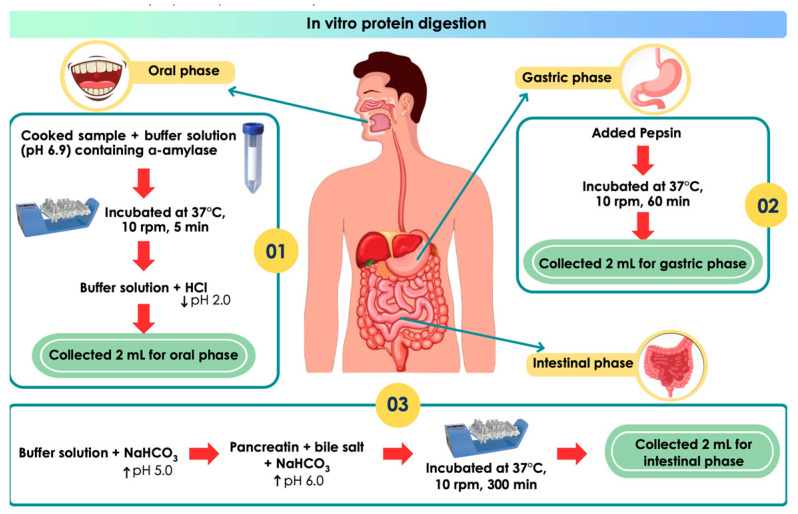
Schematic diagram illustrates in vitro protein digestion protocol.

In vitro protein digestion was carried out according to the method described in a study by Trithavisup et al. [41] and is summarized in Figure 1. All steps of the enzymatic digestion were performed at 37 °C with constant stirring (10 rpm) using a digital TRAYSTER (IKA Worke). In brief, two grams (dry basis) of ground cooked meat were mixed with 4 mL of buffer solution (120 mM NaCl, 5 mM KCl, and 6 mM CaCl_2_, pH 6.9) containing 75 U/mL α-amylase. The mixture was incubated for 5 min. Subsequently, the buffer (8 mL) was added to the mixture and the pH of the mixture was immediately reduced to pH 2.0 using 6N HCl. Pepsin was then added for a final concentration of 2000 U/mL. The mixture was then incubated for 60 min to mimic digestion in the gastric phase. Upon completion of this phase, 8 mL of the buffer were added and the pH of the mixture was then adjusted to pH 5.0 by adding 1.5 M NaHCO_3_. Solutions of pancreatin and bile salt were added to the mixture for final concentrations of 100 U/mL (based on trypsin) and 10 mM, respectively. The pH of the mixture was then adjusted to pH 6.0 using 1.5 M NaHCO_3_. The pancreatic reaction was carried out for 300 min. At the end of each phase, 2 mL of the mixture were collected. The pH of the collected mixture was adjusted to stop the enzymatic reaction before centrifugation at 3000× *g* for 30 min. Supernatant was filtered through a 0.2 μm membrane. The filtrate was used to determine free NH_2_ using the trinitrobenzenesulfonic (TNBS) acid method and free amino acid composition.

### 2.7. Free Amino (NH_2_) Content

The free amino (NH_2_) content of the supernatant collected during in vitro protein digestion was determined via the trinitrobenzenesulfonic (TNBS) acid method [42]. Briefly, the supernatants collected at the end of each digestion phase were mixed with 1% SDS in a ratio of 1:200. The reaction was then set on a 96-well plate by mixing 15 µL of sample solution, 45 µL of 0.21 M phosphate buffer (pH 8.2), and 45 µL of 0.05% *w*/*v* TNBS solution, followed by incubation at 50 °C for 1 h. Ninety microliters of 0.1 N HCl were subsequently added to each well to stop the reaction. The absorbance at 340 nm was measured using a SpectraMax Plus 384 microplate reader (Molecular Devices, San Jose, CA, USA). The standard curve was constructed using leucine as a standard solution. The free NH_2_ content was expressed as µmol per gram protein. The assay was performed in duplicate. The degree of protein hydrolysis (%DH) was calculated using the following equations [41].

(1) $$\%DH=\frac{h}{h_{tot}}\times 100\%$$

(2) $$h=\frac{\left(leucine\;{NH}_{2}-\beta \right)}{\alpha }$$

where α = 1.00, β = 0.40 [43], and h_tot_ = 7.6 mmoL/g protein [44].

### 2.8. Amino Acid Composition

Amino acid compositions in cooked breast samples was assessed using gas chromatography–mass spectrometry (GC-MS) [45]. In brief, 40 mg of the sample were hydrolyzed using 5 mL of 6 N HCl at 110 °C for 18 h. The hydrolysates were neutralized using sodium bicarbonate solution and mixed with internal standard (50 µL of norleucine). The mixture was then dried and derivatized with N-tert-butyldimethylsilyl-N-methyltrifluoroacetamide with 1% tert-butyldimethylchlorosilane. The derivatization was carried out at 100 °C for 4 h. The derivatized samples were analyzed using a 7890A GC/5975C MS (Agilent Technologies, Santa Clara, CA, USA) equipped with a DB-5 column (30 m × 0.25 mm initial diameter × 0.1 µm thickness, Agilent Technologies). The sample was injected with a split mode into the column. Helium was used as a carrier gas. The oven condition was set as follows: initial temperature 170 °C to 200 °C, hold 3 min, final increment 200 °C to 285 °C, ramp rate 4 °C/min. As for the MS condition, the temperatures of the transfer line and the ion source were set to 300 °C and 230 °C, respectively. The scan range was 35 to 800 *m*/*z*. The essential amino acid content was expressed in the unit of mg per g sample and used for the calculation of the in vitro PDCAAS.

### 2.9. Free Amino Acids

The supernatant collected at the end of the intestinal phase was profiled for free amino acids using GC-MS following a method described elsewhere [45]. Prior to the analysis, 400 mg of the supernatant were mixed with 4 mL of 25% acetonitrile in 0.1N HCl and sonicated at room temperature for 20 min. The mixture was then centrifuged at 9000 rpm for 20 min, and 150 µL of the resulting supernatant from this step was then subjected to derivatization and GC-MS analysis using a 7890A GC/5975C MS (Agilent Technologies) following the condition described for amino acid composition profiling (Section 2.6). The content of free amino acids was calculated and expressed as milligrams per 100 g of the sample.

### 2.10. In Vitro Protein Digestibility-Corrected Amino Acid Score

The in vitro PDCAASs of the samples were estimated according to the method previously described [37]. First, the amino acid score and limiting essential amino acids (EAAs) in each treatment group were identified based on the FAO/WHO recommendation [46]. In brief, the amino acid score was calculated as the ratio of amino acid content (mg/g testing food protein) in cooked meat (obtained from Section 2.6) to the FAO/WHO-recommended reference amino acid profile for three different age groups (i.e., pre-school children, school children, and adults) [46]. The EAA with the lowest ratio was identified as the limiting EAA. Subsequently, in vitro PDCAAS values were calculated as follows.

(3) $$In\;vitro\;PDCAAS=amino\;acid\;score\left(of\;the\;limiting\;EAA\right)\times \%DH$$

where the reference EAA pattern is the amino acid requirement for three different age groups, including pre-school children (2–5 years old), school children (10–12 years old), and adults (>18 years).

### 2.11. Statistical Analysis

The effects of growth-related myopathies were assessed using one-way analysis of variance (ANOVA). Prior to one-way ANOVA, the assumptions regarding homogeneity of variance and normal distribution were tested. All datasets followed those assumptions. Mean differences were subsequently analyzed using the Tukey HSD. The significant level was set at *p* < 0.05.

## 3. Results and Discussions

### 3.1. Protein Content and Cooking Loss

**Figure 2:**
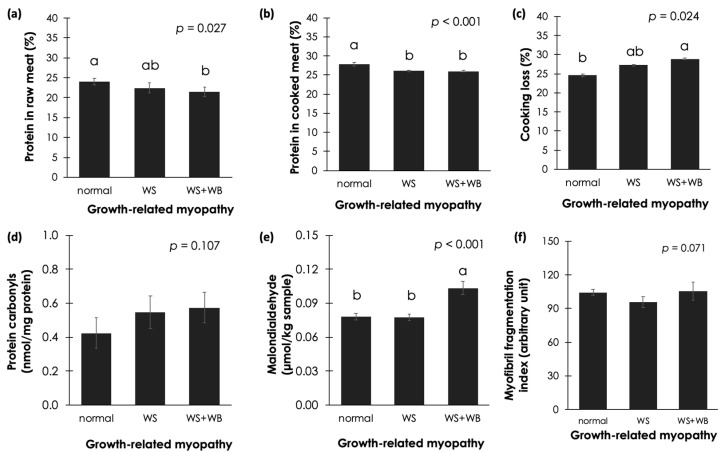
Effects of white striping (WS) and wooden breast (WB) on protein, cooking loss, and oxidation of protein and lipids in chicken breast meat. Bars and error bars represent mean and standard deviation, respectively, of (**a**) protein content in raw breast samples, (**b**) protein content in cooked breast samples, (**c**) cooking loss, (**d**) protein oxidation, (**e**) lipid oxidation, and (**f**) myofibril fragmentation index (MFI) in chicken breast meat. Different letters above the bars indicate significant difference (*p* < 0.05).

As shown in Figure 2a–c, chicken breasts with WS + WB abnormality exhibited lower protein content and greater cooking loss than those of normal samples (*p* < 0.05). As for the raw sample with WS condition alone, no differences in protein content or cooking loss were demonstrated in either normal or WS + WB samples (*p* ≥ 0.05). However, once cooked, the WS samples showed significantly lower protein than the normal samples (*p* < 0.05).

### 3.2. Oxidation of Protein

The effects of growth-related myopathies on protein oxidation in raw chicken meat were determined by monitoring the protein carbonyl content. As shown in Figure 2d, even though such values tended to be higher in the abnormal samples, no significant differences in protein carbonyls were found among the treatments (*p* ≥ 0.05).

### 3.3. Oxidation of Lipid

Considering lipid oxidation among the raw meat samples (Figure 2e), the WS + WB samples showed the highest TBARS values (*p* < 0.05). As for normal and WS samples, no significant differences were observed between the two (*p* ≥ 0.05). The results indicate that lipid oxidation occurred in the WS + WB samples to a greater extent compared to the other samples.

### 3.4. Myofibril Fragmentation Index

The effects of growth-related myopathies on the MFI are shown in Figure 2f. Although the MFI value for the WS samples tended to be lower than for the others, no significant differences in MFI were found among the treatments (*p* ≥ 0.05).

### 3.5. In Vitro Protein Digestibility

**Figure 3:**
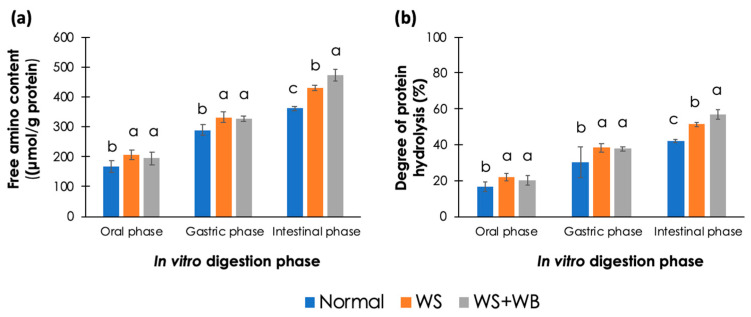
Effects of white striping (WS) and wooden breast (WB) on (**a**) free amino (NH_2_) content and (**b**) degree of protein hydrolysis (%) from in vitro protein digestion of cooked chicken breasts. Bars and error bars illustrate mean and standard deviation, respectively. Different letters above the bars indicate statistical difference (*p* < 0.05) due to growth-related myopathies at each in vitro digestion phase.

As shown in Figure 3, at all digestion phases, free NH_2_ and %DH of WS and WS + WB samples were greater than those of normal samples (*p* < 0.05). An increase in the free NH_2_ group indicated a greater hydrolysis of peptide bonds, resulting in an exposure of the NH_2_ group. The results indicate that the NH_2_ group was exposed to a greater extent in the abnormal samples.

### 3.6. Free Amino Acids Released during In Vitro Protein Digestion

**Figure 4:**
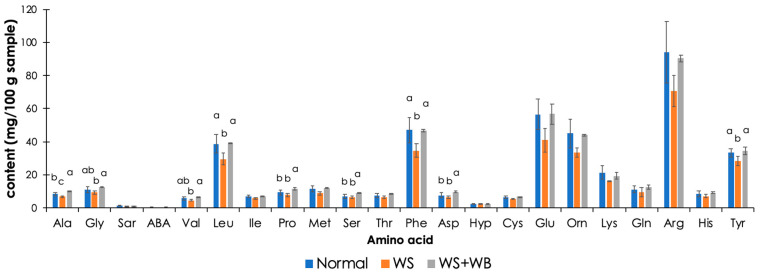
Effects of white striping (WS) and wooden breast (WB) on amino acid compositions released from digested cooked chicken breasts during in vitro protein digestion. Bars and error bars illustrate mean and standard deviation, respectively. Different letters above the bars indicate statistical difference (*p* < 0.05) due to growth-related myopathies at each in vitro digestion phase.

Development of the growth-related myopathies significantly affected the release of nine amino acids during in vitro protein digestion (Figure 4). The WS samples exhibited the lowest alanine, glycine, valine, leucine, phenylalanine, and tyrosine levels of the treatments (*p <* 0.05). The content of released glycine, valine proline, serine, and aspartic/aspartate in the WS samples did not significantly differ between normal and WS samples (*p* ≥ 0.05). In contrast, the WS + WB samples exhibited the highest alanine, proline, serine, and aspartic/aspartate levels (*p <* 0.05).

### 3.7. Essential Amino Acids and Amino Acid Scores in Cooked Chicken Breast

Essential amino acids (EAAs) were defined in the cooked breast samples (Table 1). The amino acid score was then calculated using the reference amino acid composition recommended by the FAO/WHO for three different consumer groups [46], as shown in Table 2. The results showed that the development of WS and WS + WB affected isoleucine, lysine, sulfur-containing amino acids (i.e., methionine + cysteine), aromatic amino acids (i.e., phenylalanine + tyrosine), and threonine in cooked chicken breast (Table 1). As per the amino acid scores (Table 2), for pre-school children (2–5 years of age), the limiting EAA in normal and WS breast meat was lysine. However, when the meat was affected by the WS + WB condition, the limiting EAA was shifted to aromatic amino acids (i.e., phenylalanine + tyrosine). The limiting EAA in all chicken breast samples calculated for school children (10–12 years old) was lysine. For adults, the limiting EAA in normal and WS + WB samples was revealed as histidine, but for the WS samples, lysine was identified.

### 3.8. In Vitro Protein Digestibility-Corrected Amino Acid Score

According to the limiting EAAs identified in Table 2 and the %DH obtained from in vitro protein digestion, the in vitro PDCAAS values of chicken breast meat with or without growth-related myopathies were estimated for individuals in each age group, as shown in Table 3. The in vitro PDCAAS values estimated for the WS + WB samples were greater than those estimated for the normal and WS ones in the groups of pre-school children and school children (*p* < 0.05). Comparing the normal and WS samples, there were no significant differences in in vitro PDCAAS estimated for pre-school children and school children (*p* ≥ 0.05). On the other hand, the value for WS samples estimated for adults were higher than that of normal samples (*p* < 0.05).

## 4. Discussion

The high incidence of growth-related myopathies in commercial broilers has raised concerns among poultry breeders, producers, and animal scientists. This is due to a remarkable economic loss from the incidence. The affected chicken breasts consistently showed inferior technological properties, particularly low water-holding capacity and tough texture. In this study, the lower protein content, greater cooking loss, and increased lipid oxidation observed in the WS + WB samples corresponded to previous reports addressing the reduced proportion of protein along with decreased water-holding capacity in abnormal samples [11,16,21,27,47,48]. Such a phenomenon has been associated with myodegeneration due to growth-related myopathies [11,49]. Profound adverse impacts were observed when the WB condition developed [11], which might explain why raw WS samples showed no difference in protein content from normal samples.

Growing evidence has pointed out an association between growth-related myopathies with limited vascularization in conjunction with hypoxia and oxidative stress [50]. The latest condition is attributed to an accumulation of reactive oxygen species (ROS), which further induce oxidation of proteins and lipids in the affected meat [27,28,51]. In this study, the results indicating increased lipids in the WS + WB samples compared to in the normal and WS ones are in good agreement with the previous results from Li et al. [28] and Costa Filho et al. [51]. As for protein oxidation, no significant differences in protein carbonyl content were observed among the samples (*p* ≥ 0.05); however, the mean values trended upward in the WS and WS + WB samples. This observation is in contrast with our previous findings [27]. The discrepancy might be due to the large variation among the samples in this study. Another plausible explanation might be that, in this study, the whole breast was used for the investigation. In general, WS and WB lesions are distributed unevenly in the breast. The superficial area is prone to being affected to a greater extent compared with the deeper region of the breast [28]. In this regard, as the whole breast was ground before further analyses, the investigation using the entire breast sample might have diluted the adverse effects of the myopathies. Similar explanations might also apply for the current MFI results.

Considering in vitro protein digestion, the increase in the free NH_2_ group in the abnormal samples indicates that their peptide bonds were hydrolyzed to a greater extent than in the normal samples. Consequently, more NH_2_ groups were exposed. The results correspond well with our recent investigation between non-WB and WB samples [33]. Focusing on the oral phase alone, the free NH_2_ content of both the WS and the WS + WB samples was still greater than that of the normal samples (*p* < 0.05). Since there was no proteolytic activity at this phase, the current results strongly indicate a greater degree of protein degradation within the abnormal samples [26]. Interestingly, the free NH_2_ content and the %DH of the WS and WS + WB samples at the oral and gastric phases did not differ from each other (*p* ≥ 0.05). On the other hand, at the intestinal phase, those values in the WS samples were lower than those in the WS + WB samples. Although further investigation is required, the findings might imply that the WS + WB samples were more susceptible to intestinal proteolytic activity than the meat with the WS condition alone.

Previous studies mainly addressed the effects of growth-related myopathies on amino acid composition in raw chicken breast [27]. Those previous investigations aimed at a better understanding of biological pathways associated with development of the myopathies. The significant changes in such compositions were again linked with muscle degeneration and the re-routing of metabolic pathways [26,49,52,53]. In contrast, our ultimate goal was to determine whether the growth-related myopathies impacted the protein quality of the chicken meat. In this regard, amino acid composition was analyzed in cooked chicken breasts. Two types of samples, i.e., ground cooked samples and the supernatant resulting from in vitro protein digestion, were used. Considering free amino acids released during in vitro digestion, the current results imply the reduced availability of essential amino acids, particularly valine, leucine, phenylalanine, and tyrosine, in WS chicken breast meat.

To further elucidate the effects of growth-related myopathies on the quality of proteins in chicken breast meat, the essential amino acids were characterized in cooked breast samples and used for calculation of the in vitro PDCAAS. The obtained data are in good agreement with the amino acid content of skinless, boneless chicken breast recommended by the USDA [54]. Nonetheless, the effects of growth-related myopathies on amino acid composition in the cooked meat differed from the profile of free amino acids released during in vitro protein digestion, as shown in Section 3.6. The explanation requires further investigation. The findings, however, further affirm that, upon development of the WS and WS + WB conditions, essential amino acids, particularly isoleucine, sulfur-containing amino acids, and aromatic amino acids, tended to decrease in the abnormal chicken breast meat. According to the greater estimated in vitro PDCAAS values in the abnormal samples, the current results suggest that growth-related myopathies tended to increase protein digestibility in the cooked meat. However, the shifts in limiting EAAs in chicken breast meat due to the development of WS and WB are still worth underlining.

One important note should be discussed herein. In general, animal-derived foods have a PDCAAS value equivalent to or slightly below 100% [55]. However, the estimated in vitro PDCAAS values obtained from this study were below 50%. The discrepancies could be due to the low protein digestibility (%DH < 50) used in the calculation. The current %DH is in good agreement with the previously obtained dataset [33,41]. We previously compared the in vitro protein digestion method for chicken meat between the standard protocol INFOGEST [56] and the method described by Bordoni et al. [57]. Indeed, the %DH from both methods was below 50%, but the latter protocol resulted in a greater %DH for cooked chicken breast [41]. Therefore, we adopted the method by Bordoni et al. [57] for our experiments. Another limitation of this in vitro protein study was that the results could not elucidate the effects of growth-related myopathies on protein absorption or metabolic availability of the chicken meat protein. The current findings underline the limitation of the in vitro method for evaluating dietary protein quality. Nonetheless, the obtained results offer an insight regarding the effects of growth-related myopathies on in vitro protein digestion and in vitro PDCAAS of cooked chicken breast meat.

Dietary requirements of amino acids and proteins vary among individuals based on several factors, including dietary factors, physiological characteristics (e.g., age, sex, genetic background, and physical activities), pathological conditions (e.g., infection, obesity, and diabetes), and environmental factors (e.g., pollution, toxic exposure, and personal hygiene) [58]. Therefore, it is crucial to consider all factors when defining dietary amino acid requirements. The deficiency of dietary proteins contributes to poor growth, particularly in children, and often affects biological mechanisms in terms of the absorption and transportation of other nutrients (e.g., vitamin A and iron). As a consequence, protein deficiency can worsen the deficiency of other nutrients. On the contrary, excessive protein intake can overwhelm the capability of the liver to remove toxic protein metabolites, particularly ammonia [58]. In this regard, it is strongly recommended that the amount of dietary protein intake be carefully monitored.

Overall, the current findings suggest that cooked chicken breast affected by the WS + WB condition might provide greater protein digestibility and availability than breasts with WS and normal breasts. Regardless, because of the lower protein content and shifts in limiting EAAs in the growth-related myopathies, the consumption of chicken breast meat might need a revisit by all adhering bodies (e.g., nutritionists, dietitians, and food scientists). In addition, further in-depth investigation is indeed required to ensure protein quantity and quality for all groups of consumers.

## 5. Conclusions

In conclusion, the current study shows that cooked WS and WS + WB chicken breasts were more susceptible to proteolytic activities during in vitro protein digestion. The shifts in limiting EAAs for the adult and pre-school child groups were observed for cooked WS and WS + WB meats, respectively. The in vitro PDCAAS values of the WS + WB samples were greater than those of the other samples for pre-school children, school children, and adults (*p* < 0.05). Based on the obtained in vitro PDCAAS values alone, the results suggest that the WS + WB samples appeared to offer greater values of protein digestibility and availability compared to WS and normal chicken breast. Other safety aspects indeed remain to be examined to ensure no adverse health impacts from consuming chicken with growth-related myopathies.

## Figures and Tables

**Table 1:** Essential amino acids (mg/g testing food protein) in cooked chicken breasts.

Essential Amino Acid	Normal	WS	WS + WB	*p*-Value
Histidine	12.55 ± 0.39	12.29 ± 0.78	12.03 ± 0.68	0.94
Isoleucine	13.09 ^a^ ± 0.34	10.24 ^b^ ± 0.66	11.20 ^ab^ ± 1.02	*0.01*
Leucine	22.96 ± 0.48	21.05 ± 1.25	20.96 ± 1.45	0.25
Lysine	13.02 ^b^ ± 1.10	11.62 ^b^ ± 0.36	16.99 ^a^ ± 0.41	*0.002*
Methionine + cysteine	23.03 ^a^ ± 1.67	21.43 ^a^ ± 1.24	14.08 ^b^ ± 1.90	*0.002*
Phenylalanine + tyrosine	19.43 ^a^ ± 0.31	17.47 ^b^ ± 0.56	17.46 ^ab^ ± 0.87	*0.03*
Threonine	15.47 ^a^ ± 0.12	14.38 ^b^ ± 0.33	14.20 ^b^ ± 0.34	*0.02*
Valine	13.94 ± 0.37	12.07 ± 0.91	12.29 ± 0.72	0.06

Mean ± standard deviation. The *p*-value in italics indicates significance for *p* < 0.05. Different superscripts within the same row indicate statistical differences (*p* < 0.05).

**Table 2:** Amino acid score estimated for cooked chicken breast meat with or without growth-related myopathies.

Essential Amino Acid	Reference ^1^ (mg/g Food Protein)	Normal	WS	WS + WB
*Pre-school children (2–5 years old)*				
Histidine	19	0.66 ± 0.02	0.65 ± 0.04	0.63 ± 0.04
Isoleucine	28	0.47 ± 0.01	0.37 ± 0.02	0.40 ± 0.04
Leucine	66	0.35 ± 0.01	0.32 ± 0.02	0.32 ± 0.02
Lysine	58	**0.22 ± 0.02**	**0.20 ± 0.01**	0.29 ± 0.01
Methionine + cysteine	25	0.92 ± 0.27	0.86 ± 0.05	0.56 ± 0.08
Phenylalanine + tyrosine	63	0.31 ± 0.00	0.28 ± 0.01	**0.28 ± 0.01**
Threonine	34	0.45 ± 0.00	0.42 ± 0.01	0.42 ± 0.01
Valine	35	0.40 ± 0.01	0.34 ± 0.03	0.35 ± 0.02
*School children (10–12 years old)*				
Histidine	19	0.66 ± 0.02	0.65 ± 0.04	0.63 ± 0.04
Isoleucine	28	0.47 ± 0.01	0.37 ± 0.02	0.40 ± 0.04
Leucine	44	0.52 ± 0.01	0.48 ± 0.03	0.48 ± 0.03
Lysine	44	**0.30 ± 0.02**	**0.26 ± 0.01**	**0.39 ± 0.01**
Methionine + cysteine	22	1.05 ± 0.08	0.97 ± 0.06	0.64 ± 0.09
Phenylalanine + tyrosine	22	0.88 ± 0.01	0.79 ± 0.03	0.79 ± 0.04
Threonine	28	0.55 ± 0.00	0.51 ± 0.01	0.51 ± 0.01
Valine	25	0.56 ± 0.01	0.48 ± 0.04	0.49 ± 0.13
*Adults (>18 years old)*				
Histidine	16	**0.78 ± 0.02**	0.77 ± 0.05	**0.75 ± 0.04**
Isoleucine	13	1.01 ± 0.03	0.79 ± 0.05	0.86 ± 0.08
Leucine	19	1.21 ± 0.03	1.11 ± 0.07	1.10 ± 0.08
Lysine	16	0.81 ± 0.07	**0.73 ± 0.02**	1.06 ± 0.03
Methionine + cysteine	17	1.35 ± 0.10	1.26 ± 0.07	0.83 ± 0.11
Phenylalanine + tyrosine	19	1.02 ± 0.02	0.92 ± 0.03	0.92 ± 0.05
Threonine	9	1.72 ± 0.01	1.60 ± 0.04	1.58 ± 0.04
Valine	13	1.07 ± 0.03	0.93 ± 0.07	0.95 ± 0.06

^1^ FAO/WHO amino acid reference pattern [46] for three different age groups. Mean ± standard deviation. The number in bold indicates the lowest amino acid score.

**Table 3:** Limiting essential amino acid (EAA) and in vitro protein digestibility-corrected amino acid score (PDCAAS) of chicken breast meat for three different consumer age groups.

Parameter	Normal	WS	WS + WB
***Pre-school children*** *(2–5 years old)*			
Limiting EAA	Lysine	Lysine	Phenylalanine + tyrosine
Amino acid score	0.22 ± 0.02	0.20 ± 0.01	0.28 ± 0.01
In vitro protein digestion (%)	42.32 ± 0.96	51.42 ± 1.24	57.02 ± 2.61
In vitro PDCAAS (%)	9.50 ^b^ ± 0.80	10.31 ^b^ ± 0.32	15.80 ^a^ ± 0.79
***School children*** *(10* *–12 years old)*			
Limiting EAA	Lysine	Lysine	Lysine
Amino acid score	0.30 ± 0.02	0.26 ± 0.01	0.39 ± 0.01
In vitro protein digestion (%)	42.32 ± 0.96	51.42 ± 1.24	57.02 ± 2.61
In vitro PDCAAS (%)	12.53 ^b^ ± 1.05	13.58 ^b^ ± 0.43	22.02 ^a^ ± 0.53
***Adults*** *(>18 years old)*			
Limiting EAA	Histidine	Lysine	Histidine
Amino acid score	0.78 ± 0.02	0.73 ± 0.02	0.75 ± 0.04
In vitro protein digestion (%)	42.32 ± 0.96	51.42 ± 1.24	57.02 ± 2.61
In vitro PDCAAS (%)	33.20 ^c^ ± 1.04	37.36 ^b^ ± 1.17	42.89 ^a^ ± 2.43

EAA = essential amino acid. Mean ± standard deviation. Different superscripts depict significant difference in the in vitro PDCAAS due to growth-related myopathies within the same age group (*p* < 0.05).

## Data Availability

Data is contained within the article.
